# Neovascularization of coronary *tunica intima* (DIT) is the cause of coronary atherosclerosis. Lipoproteins invade coronary intima via neovascularization from adventitial *vasa vasorum*, but not from the arterial lumen: a hypothesis

**DOI:** 10.1186/1742-4682-9-11

**Published:** 2012-04-10

**Authors:** Vladimir M Subbotin

**Affiliations:** 1 602 Samuel Drive, Madison, WI, 53717, USA

## Abstract

**Background:**

An accepted hypothesis states that coronary atherosclerosis (CA) is initiated by endothelial dysfunction due to inflammation and high levels of LDL-C, followed by deposition of lipids and macrophages from the luminal blood into the arterial intima, resulting in plaque formation. The success of statins in preventing CA promised much for extended protection and effective therapeutics. However, stalled progress in pharmaceutical treatment gives a good reason to review logical properties of the hypothesis underlining our efforts, and to reconsider whether our perception of CA is consistent with facts about the normal and diseased coronary artery.

**Analysis:**

To begin with, it must be noted that the normal coronary *intima* is not a single-layer endothelium covering a thin acellular compartment, as claimed in most publications, but always appears as a multi-layer cellular compartment, or diffuse intimal thickening (DIT), in which cells are arranged in many layers. If low density lipoprotein cholesterol (LDL-C) invades the DIT from the coronary lumen, the initial depositions ought to be most proximal to blood, i.e. in the inner DIT. The facts show that the opposite is true, and lipids are initially deposited in the outer DIT. This contradiction is resolved by observing that the normal DIT is always avascular, receiving nutrients by diffusion from the lumen, whereas in CA the outer DIT is always neovascularized from adventitial *vasa vasorum*. The proteoglycan biglycan, confined to the outer DIT in both normal and diseased coronary arteries, has high binding capacity for LDL-C. However, the normal DIT is avascular and biglycan-LDL-C interactions are prevented by diffusion distance and LDL-C size (20 nm), whereas in CA, biglycan in the outer DIT can extract lipoproteins by direct contact with the blood. These facts lead to the single simplest explanation of all observations: (1) lipid deposition is initially localized in the outer DIT; (2) CA often develops at high blood LDL-C levels; (3) apparent CA can develop at lowered blood LDL-C levels. This mechanism is not unique to the coronary artery: for instance, the normally avascular cornea accumulates lipoproteins after neovascularization, resulting in lipid keratopathy.

**Hypothesis:**

Neovascularization of the normally avascular coronary DIT by permeable vasculature from the adventitial *vasa vasorum* is the cause of LDL deposition and CA. DIT enlargement, seen in early CA and aging, causes hypoxia of the outer DIT and induces neovascularization. According to this alternative proposal, coronary atherosclerosis is not related to inflammation and can occur in individuals with normal circulating levels of LDL, consistent with research findings.

## Background

Atherosclerosis, the predominant cause of coronary artery disease, remains enigmatic. Despite best efforts, available therapies protect only 30-40% of individuals at risk, and no therapeutic cure is anticipated for those who currently suffer from the disease. Delayed progress concerning pharmaceutical treatment implies that atherosclerosis drug development is in jeopardy, raising concerns among experts [1].

This analysis addresses the logical properties of the hypothesis underlying our efforts, and reconsiders whether our perception of the disease is consistent with undisputed facts concerning coronary arteries in general and during disease in particular. A different perspective on the pathogenesis of atherosclerosis is proposed.

## Logical properties and factual consistency concerning a currently endorsed hypothesis relating to coronary atherosclerosis: common perception of coronary artery morphology

**Figure 1:**
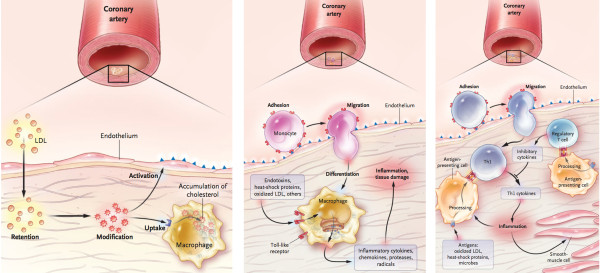
**From: Hansson GK. Inflammation, atherosclerosis, and coronary artery disease. ***The New England journal of medicine* 2005; 352(16):1685–1695. Figures 2,3 and 4[5]. Reproduced with permission of the Publisher. Copyright © MMS, 2005.

A currently endorsed hypothesis is based on the following assumptions: (1) atherosclerosis is a systemic disease, initiated by endothelial dysfunction due to (2) inflammation and (3) high levels of LDL, (4) leading to lipid and macrophage deposition in the *tunica intima* from blood of the coronary lumen, and plaque formation (modified response-to-injury hypothesis) [2,3]. This perception is presented in mainstream scientific publications and in educational materials, whether printed or electronic. This hypothesis is typically accompanied by familiar schematics depicting the pathogenesis of coronary atherosclerosis and transition from a normal cardiac artery to a diseased state, e.g. Figure 1:

This perception of the mechanism of disease and similar schematics appear in well-recognized scientific journals including Nature Medicine, Atherosclerosis, Thrombosis and Vascular Biology and etc. (e.g. [5]), and common educational materials such as the Britannica Online Encyclopaedia:

Therefore, this explanatory model concerning atherosclerosis, and accompanying schematics indistinguishable from that outlined above, are available in the majority of scientific publications and educational materials [2-6].

## Analysis of main assumptions of the currently endorsed hypothesis

### Assumption: atherosclerosis is a systemic disease

#### Factual contradiction

Atherosclerosis never affects the entire arterial bed; it is exclusive to large muscular arteries, particularly coronary, and to a lesser extent to elastic arteries. Therefore, this systemic notion should be rejected on logical grounds; atherosclerosis is NOT a systemic disease.

### Assumption: atherosclerosis is an inflammatory disease

Varieties of microorganisms are present in advanced atherosclerotic lesions, for example in specimens removed during atherectomy [7]. Fabricant *et al*. induced visible atherosclerotic changes in chicken coronary arteries resembling that in humans, by infecting them with herpesvirus [8-10] and suggested the viral role in pathogenesis, a view shared by many scientists (for review see [11,12]). *Mycoplasma pneumonia* or *Chlamydia pneumoniae* infections alone [13] or together with influenza virus [14] have been proposed as contributory factors in the pathogenesis of atherosclerosis, and particularly by participation in obstruction of *vasa vasorum*[11]. However, these cases probably do not indicate the initiation of atherosclerosis, but are more likely to represent secondary infection of degenerating/necrotic tissue. It should be emphasized that neither non-steroidal nor antibacterial anti-inflammatory treatments alter the risk of coronary atherosclerosis [15-18]. Despite the aforementioned studies [7-11,13,14], therefore, it can reasonably be claimed that no infectious cause of atherosclerosis has been demonstrated [19,20].

### Assumption: a high level of LDL initiates and is the main cause of atherosclerosis

High levels of LDL are an important risk factor, and lowering LDL levels is the most significant pharmaceutical tool in coronary atherosclerosis prevention. However, the statement that high levels of LDL are the main cause of coronary atherosclerosis is inconsistent with established medical concepts.

#### Inconsistency with the established concept in medicine

“Indeed, proof that a given condition always precedes or accompanies a phenomenon does not warrant concluding with certainty that a given condition is the immediate cause of that phenomenon. It must still be established that when this condition is removed, the phenomenon will no longer appear….” Claude Bernard [21].

As has been emphasized by numerous scientists, multiple factors participate during disease development, and can affect the progression and severity of disease. However, only through distinguishing the cause from all contributing factors can an effective cure, leading to disease eradication, be achieved.

"“… differentiating between cause and non-causative factors is essential. Elimination of the latter only ameliorates or reduces the incidence whereas elimination of the former eradicates the disease. Swamps are not a cause of malaria. Draining swamps may reduce the incidence of malaria but it is eradication of the malarial parasites that eliminates the disease. Reduction in incidence rather than elimination of the disease precludes a causal relationship.” W. E. Stehbens [22]."

Therefore, the fact that lowering LDL levels does not prevent cardiac events in 60-70% of individuals at risk [23] contradicts the causative role of LDL. Unfortunately, it appears that the scientific and medical communities are focusing on and emphasizing biomarkers that can predict risk, without proof that these biomarkers cause the risk [24,25].

Mechanisms of diseases constitute a new scientific field. However, although well-recognized concepts are not always proved correct, the author believes that a new hypothesis should not contradict established concepts that have been proven as far as possible, without informed reasoning.

#### Factual discrepancies

Lipid/macrophage pathogenesis of arteriosclerosis was suggested approximately one hundred years ago [26]. However, the hypothesis only gained proper attention during the 1970-80s, after a report concerning the Framingham Heart Study [27], culminating in joint NIH and American Heart Association publication of a Special Report [28], which was reprinted in all relevant journals [29-33]. The first Panel’s Conclusion of the Report states: “Elevation of blood cholesterol levels is a major cause of coronary artery disease”.

At approximately the same time, effective hypolipidemic drugs were developed and introduced to clinics, and the American Heart Association predicted that lowering blood cholesterol would almost eliminate the requirement for bypass surgery and eradicate coronary arteriosclerosis by the end of the 20^th^ century [5,34]. It is now known that HMG-CoA reductase inhibitors, cholesterol-lowering drugs known as “statins”, are almost 100% effective in populations with high LDL-C levels, but normalizing LDL levels only reduces the risk of cardio-vascular diseases in this group by approximately 30-40% [23,35-38], and the total number of coronary interventions (bypass and stenting operations) has increased significantly [39]. However, individuals with normal LDL-C levels suffer from coronary atherosclerosis, and although at lower risk, this includes vegetarians [40]. Numerous studies have demonstrated that coronary atherosclerosis affects all *eutherian* animals with a body mass comparable to or larger than humans, regardless of diet specialization and LDL levels [41-45]. Surprisingly, in these *mammals*, lipid accumulations in arterial walls were more common in herbivores than carnivores [43,46]. The lack of association between total or LDL cholesterol and degree of atherosclerosis in unselected individuals was demonstrated by a study during the 1930s [47] and has since been noted by many others, notably by W. E. Stehbens [48-54] and U. Ravnskov [55-59], and others, e.g. [60]. Therefore, the hypothesis that elevated blood cholesterol constitutes a major cause of coronary arteriosclerosis is questionable. Undoubtedly, high LDL levels are an important risk factor and a vital tool in CA prevention, but logically, it must be concluded that high LDL levels are not "a major cause" of coronary atherosclerosis.

### Assumption: lipids act and invade coronary *tunica intima* from the arterial lumen

#### Factual discrepancies

If high levels of LDL-C affect and invade arterial walls from the arterial lumen (Figure 1), then the initial and most pronounced lipid accumulation in the arterial *tunica intima* ought to be most proximal to the coronary blood flow, i.e. within inner layers of the *tunica intima*. However, detailed pathological studies concerning the early stages of human coronary atherosclerosis have demonstrated that the opposite is true, i.e. lipid deposits are initiated on outer layers of the coronary *tunica intima*[61,62], termed deeper musculoelastic layers (for morphological details and terms see [63]). A report published in 1968 described, although very briefly, the same morphological pattern during the early stages of human coronary atherosclerosis: initial lipid accumulation in the deepest intimal portion, followed by lipid deposition in the middle intimal zone [64]. This counterintuitive location of lipid depositions is very important for understanding the pathogenesis of coronary atherosclerosis, and I term this phenomenon the “outer lipid deposition paradox”.

**Figure 2:**
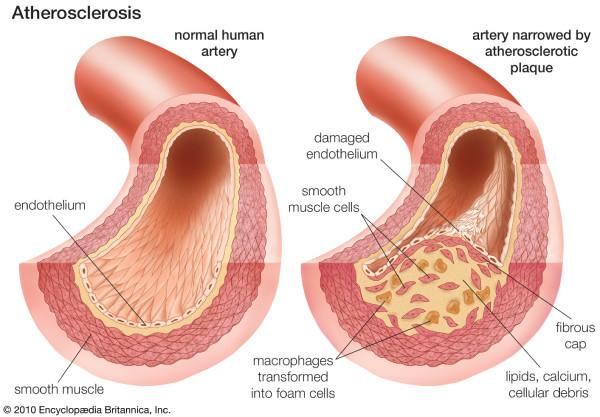
**Coronary atherosclerosis.** From: Atherosclerosis. Britannica Online Encyclopaedia. By courtesy of Encyclopaedia Britannica, Inc., copyright © 2010 Encyclopaedia Britannica, Inc; used with permission. [4].

**Figure 3:**
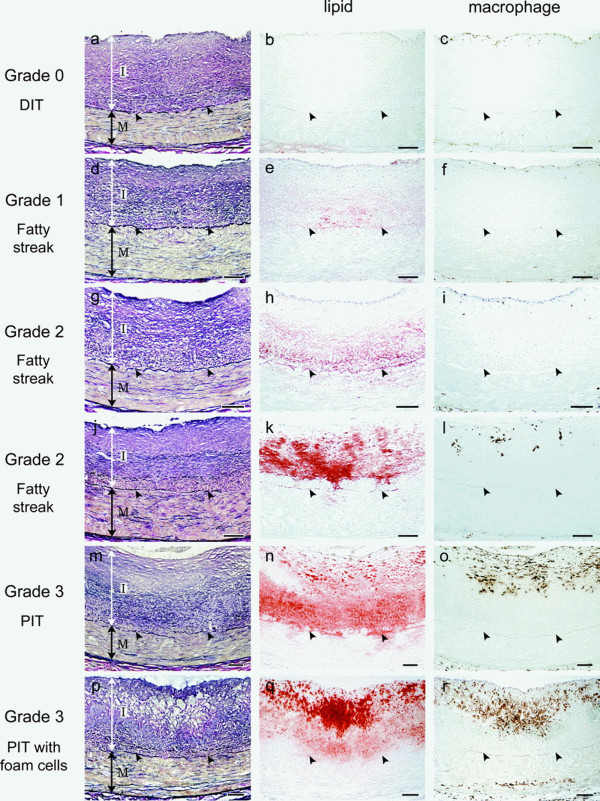
**From: Nakashima Y, Fujii H, Sumiyoshi S, Wight TN, Sueishi K.** Early human atherosclerosis: accumulation of lipid and proteoglycans in intimal thickenings followed by macrophage infiltration. *Arteriosclerosis, thrombosis, and vascular biology* 2007; 27(5):1159–1165. Arrowheads indicate internal elastic lamina. Reproduced with permission from the Publisher. Copyright © 2007, Wolters Kluwer Health.

**Figure 4:**
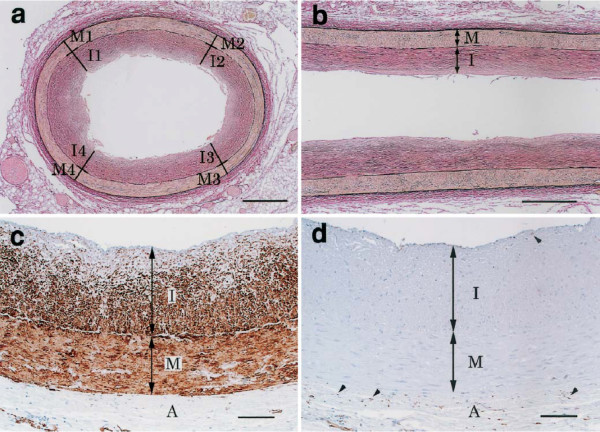
**Structures and components of DIT in the proximal portion of the RCA in adults.a**, **b** – DIT was demonstrated as a uniformly thickened inner layer (van Gieson). **c** – immunostain for alpha smooth muscle actin. Almost all cells in DIT were smooth muscle cells. **d** – immunostain for macrophage marker HAM56 at the same site as in c. Only a few intimal and several adventitial cells were positive (arrowheads). I – intima, M – media, A – adventitia. These microscopic images represent a normal right adult coronary artery in two intersecting planes. From: Nakashima Y, Chen YX, Kinukawa N, et al: Distributions of diffuse intimal thickening in human arteries: preferential expression in atherosclerosis-prone arteries from an early age. *Virchows Arch* 2002, **441**:279–288. Used with permission from the publisher and authors. Copyright © 2002, Springer.

**Figure 5:**
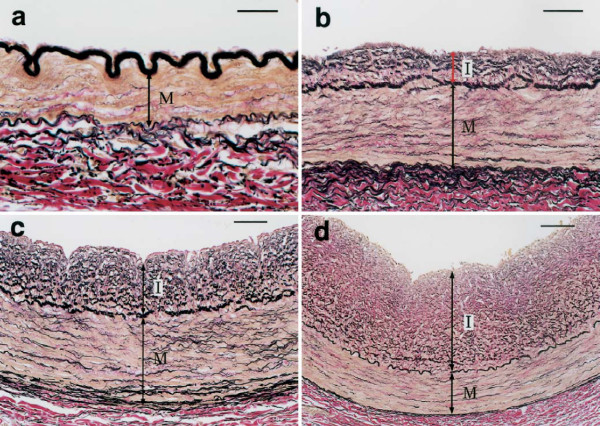
**Diffuse intimal thickening (DIT) in proximal coronary arteries.a**– Right coronary artery (RCA), 7-day-old female. **b** – Left anterior descending artery (LAD), 5-year-old female. **c** – LAD, 15-year-old female. **d** – LAD, 29-year-old female. Bars in a, b, c and d represent 25 μm, 50 μm, 50 μm, 100 μm, respectively. I – intima, M – media. These microscopic images represent normal morphological changes in coronary arteries from birth to adult (van Gieson stain). From: Nakashima Y, Chen YX, Kinukawa N, et al: Distributions of diffuse intimal thickening in human arteries: preferential expression in atherosclerosis-prone arteries from an early age. *Virchows Arch* 2002, **441**:279–288. Used with permission from the publisher and authors. Copyright © 2002, Springer.

Nakashima *et al*. explained the outer lipid deposition paradox by demonstrating that accumulation of proteoglycan biglycan occurs predominantly in the outer layers of the *tunica intima* of normal and diseased individuals, i.e. in the same location as the initial accumulation of lipids. Furthermore, Nakashima *et al*. suggested that biglycan possesses specific binding properties for atherogenic lipoproteins. They noted that structural changes in biglycan could increase its binding properties, and suggested a possible source of biglycan expression in agreement with previous reports [65,66]. Noting some discrepancy in patterning, i.e. that lipids deposit eccentrically, whereas biglycan is localized concentrically [62], the authors elaborated these specifics in this and a later publication [67].

In addition to reporting significant findings on the precise location of lipid depositions during initiation of coronary atherosclerosis, this work univocally demonstrates that normal coronary *tunica intima* is not a single-layer endothelium covering a thin acellular compartment, as is commonly claimed in all mainstream scientific publications and educational materials (e.g. Figures 1 and 2), but a multi-layer cellular compartment where cells and matrix are arranged in a few dozen layers.

However, this is not a new discovery in coronary morphology. In 2002 Nakashima *et al*. published a complete morphological analysis concerning normal post-natal development of human coronary arteries, demonstrating that the epicardial coronary *tunica intima* invariably forms a multilayered cellular compartment, or diffuse intimal thickening (DIT) [68], known as normal arterial intimal hyperplasia [69]. Please note, this morphogenesis necessarily occurs during early postnatal development in humans and is maintained throughout life.

From: Nakashima Y, Chen YX, Kinukawa N, et al: Distributions of diffuse intimal thickening in human arteries: preferential expression in atherosclerosis-prone arteries from an early age. *Virchows Arch* 2002, **441**:279–288. Used with permission from the publisher and authors. Copyright © 2002, Springer.

From: Nakashima Y, Chen YX, Kinukawa N, et al: Distributions of diffuse intimal thickening in human arteries: preferential expression in atherosclerosis-prone arteries from an early age. *Virchows Arch* 2002, **441**:279–288. Used with permission from the publisher and authors. Copyright © 2002, Springer.

Nakashima *et al*. [68] credited all previous reports concerning DIT in normal human coronaries, beginning with a famous publication by Richard Thoma in 1883 [70] and concluding with modern papers, e.g. [71]. These references could be supplemented with dozens of others demonstrating that the formation of DIT in normal coronaries is universal in humans. One particular publication, written by Dr. Kapitoline Wolkoff in 1923 [72], was pioneering in relation to the detailed morphology of post-natal human coronary ontogenesis. In her observations, the intimal structures (in German “Bindegewebsschicht” and “Elastisch-hyperplastische Schicht”) above a *lamina elastica interna* correspond to DIT in the modern literature [63,67,68,73].

To my knowledge there are no definitive data concerning the number of cell layers forming DIT, which varies in formalin-fixed specimens owing to artery contraction in fixative [63]. In addition to individual variations, the latter could explain differences in DIT thickness in various reports, e.g. [68,72,74]. Therefore, it is difficult to determine an exact number of cell layers in DIT, although extrapolating from all available reports it can be approximated as between 20–25 and 35–50 cell layers. Coronary artery DIT has been found in all studies concerning vertebrates with a body mass similar to or larger than humans (for review see [69]), and taxonomy-wise starting with fishes [75]. Unfortunately, these fundamental facts have not been widely appreciated during medical research and education, which commonly operates on the assumption that normal coronary arterial *tunica intima* is always an "ideal" single-layer endothelium covering an acellular compartment [4-6,76], or denying the presence of coronary DIT in animals [77].

## Discussion

When considering coronary atherosclerosis, we inevitably focus on atherosclerotic plaques, their vulnerability and rupture, lipid and necrotic core, fibrous cap and thickness, as these features determine morbidity and mortality. However, these are features of advanced stages of the disease, and such lesions [78-80] are extremely resistant to therapeutics. Progress in plaque stabilization and regression has been reported, but the probability that these patients will require coronary intervention is very high (for review see [81]). This analysis concerns initiation and early stages of CA, which should be more receptive to therapeutics and are potentially reversible. In addition, initial tissue transformations are more informative in terms of elucidating mechanisms of disease, as later pathological formations (e.g. mature plaque) include significant secondary lesions, which could mask crucial features of disease pathogenesis.

An important part of this analysis is devoted to the consistency of the hypothesis that guides our efforts to understand coronary atherosclerosis, relating to facts concerning normal coronary morphology and the diseased state. As demonstrated above, the morphology of human coronary arteries is not what is commonly claimed in analyses relating to coronary atherosclerosis, which underlies approaches to finding a cure. Unfortunately, this inaccurate perception of coronary artery morphology has led to hypotheses that imply that DIT is a dimensionally insignificant compartment, e.g. [4-6]. Furthermore, such depiction appears in articles that include micrographs of coronary artery histological slides that demonstrate the real ratio between coronary artery coats, e.g. [82]

Therefore, although the coronary *tunica intima* is a multi-layered cellular compartment equal to or thicker than the *tunica media*[62,63,67,68,70,72,83-85], there is a common perception that the human coronary *tunica intima* is a one-cell layer covering a thin matrix layer [4-6,82,86]. Since this perception is very persistent in scientific publications and educational materials, I believe it is worthwhile to look for a reason for this misinterpretation.

Custom replies such as: “it is just an unimportant visual (or verbal) schematic, but the foundation of the hypothesis is correct” are not convincing. A schematic that presents a hypothesis is the essence of the hypothesis. Therefore, if the schematic is incorrect, the hypothesis must be incorrect too.

Incorrect presentation of human coronary morphology (depicting the *tunica intima* as one cell layer covering a thin matrix layer) has several negative consequences, but the most crucial is that such misperception cannot incorporate the outer lipid deposition paradox. Even when early intimal lipid deposition is mentioned, incorrect presentation of *tunica intima* morphology as a one cell layer structure covering a thin matrix layer does not make outer lipid deposition surprising (paradoxical) and prevents a hypothesis from using this observation as a tool during analysis of the disease pathogenesis [82].

One plausible explanation for this oversight could be that medical scientists in mainstream research are not aware of the exact coronary artery morphology or consider it an insignificant detail. This is probably a reflection of how coronary histology is taught to medical students. Any standard textbook of histology, e.g. [87-89], and most monographs concerning coronary disease, e.g. [90-93], present coronary morphology in this way. The famous "Color Atlas of Cytology, Histology, and Microscopic Anatomy" used by medical students and published by Wolfgang Kuehnel [94], which was translated into all Western languages, does not include coronary artery morphology, leaving readers with the illusion that it has the same morphology as any artery of this caliber. At best, some textbooks comment briefly that the intima of elastic arteries may be thicker [95,96] or that the intima of coronary arteries demonstrates the greatest age-related changes [97,98], still stressing the single-cell layer intimal design. An example of such misrepresentation appears in the very popular Medscape website (a part of WebMD), which advertises itself as: “Medscape from WebMD offers specialists, primary care physicians, and other health professionals the Web's most robust and integrated medical information and educational tools” [99]. In its recently updated article relating to coronary artery atherosclerosis, Medscape states: “The healthy epicardial coronary artery consists of the following 3 layers: Intima, Media, Adventitia. The intima is an inner monolayer of endothelial cells lining the lumen; it is bound on the outside by internal elastic lamina, a fenestrated sheet of elastin fibers. The thin subendothelial space in between contains thin elastin and collagen fibers along with a few smooth muscle cells (SMCs)” [100]. The few modern textbooks presenting correct information, e.g. "Histology for Pathologist" [101] and “Vascular Pathology” [102], have not changed this common perception. Regardless of whether the above explanation is correct or not, this misperception of coronary artery design persists in research and education.

Failure to incorporate facts concerning coronary artery design into hypotheses relating to the mechanism(s) of coronary atherosclerosis is worrying. The accepted hypothesis describes lipid invasion into the coronary DIT from the arterial lumen [5,6,82,86,103,104]. The accepted vector and topology of events is the core of the hypothesis and the assumed mechanism of the disease: “Lipids enter the arterial wall as compounds with protein fractions of blood plasma directly from arterial lumen” [105]. This pathway is univocally incorporated in the currently endorsed hypothesis and all offshoot models. Logically, from these models, initial lipid deposition in the *tunica intima* should be more proximal to the lumen. However, it has been demonstrated that lipid accumulation appears not in the inner layers of DIT, which are proximal to the lumen, but in the distant outer layers [61,62,64,67]. Obviously, to reach an outer intimal layer, lipids are required to diffuse through numerous cell layers and a significant amount of matrix situated between the intimal cells. However, in diffusion or “filtration pressure” [106] models, the highest lipid accumulation must be most proximal to a lumen, diminishing proportionally to intimal depth, comparable to patterns of lipid accumulation in *tunica intima* of non-diseased human aortas of individuals aged 6–15 years [107]. Therefore, why does lipid accumulation in coronary atherosclerosis start in the deep layers of DIT, just above the internal elastic lamina, distant from the lumen? To explain this contradiction, the conventional hypothesis has to relate to certain conditions under which this puzzling pattern could be theoretically possible: e.g. co-localization of proteoglycan biglycan (which has a high binding capacity for lipoproteins) in the outer layer of DIT [62,67,82]. However, findings concerning biglycan location [62,67] could explain retention but not penetration, and even the former can only be explained with reservations: biglycan is expressed in several tissues of the body, so why is the outer DIT of coronary the target? Is this complicated model the only explanation?

Details of coronary artery structure are critically important for this analysis. Therefore, it is necessary to enumerate undisputed facts concerning coronary artery morphology. The human heart has coronary arteries in which a single-cell layer of *tunica intima* differentiates early in life to form DIT, and then continues to self-renew in a controlled manner throughout life in a majority of the population. When normal DIT becomes diseased, it is difficult to distinguish early pathology morphologically from the norm [108,109], and sometimes this is the case with advanced stages (post-transplant coronary atherosclerosis) [76]. Normal DIT, or normal intimal hyperplasia, is so striking in its resemblance to diseased hyperplasia that the former is known as "benign intimal hyperplasia" [110-112].

It is important to highlight that normal human coronary *tunica intima*, evolving from one cell-layer after birth to DIT in adults, is always the avascular compartment and remains avascular in the vast majority of hearts throughout life. Several studies have investigated this topic thoroughly and concluded that coronary *tunica intima* receives oxygen and nutrients through diffusion from the arterial lumen [106,113-116]; a previous suggestion that nutrients from *vasa vasorum* can meaningfully contribute to coronary *tunica intima* nourishment [117] was never confirmed. Past findings concerning the vasculature in normal coronary intima [118], later reprinted in [119], were attributed to high pressure of injected dye (ten times higher than normal) [106]. Therefore, when DIT attains thickness of up to ten cell layers (at approximately five years old), inner and outer compartments of tunica intima are exposed to various concentrations of blood constituents, as diffusion is inversely proportional to the square of the distance (i.e. DIT thickness). When this distance is increased, as happens in adult coronary DIT, it must be assumed that contact of outer intimal layers with certain blood constituents would be significantly minimized, if not completely diminished. Therefore, for adult or aged-thickened [120] and diseased-thickened coronary *tunica intima*, diffusion deficit of the outer intimal layers can be assumed, similar to the model of Wolinsky and Glagov, known as “critical depth” of avascular media or “rule 29” [121].

As aforementioned, before plaque formation occurs, diseased DIT, or pathologic intimal thickening (PIT), is microscopically indistinguishable from normal DIT. However, there is one characteristic that distinguishes diseased coronary DIT from normal DIT: pathological DIT (PIT), even during the beginning of the disease, is always vascularized [106,113-115,122]. This neovascularization, originating from adventitial *vasa vasorum*[123,124], is observed prior to the appearance of any atherosclerotic features except an increased dimension of DIT [125]. This neovascularization pattern is common in all diseased arterial DIT [126]. Contrary to a previous report concerning coronary atherosclerosis [118,119], in contemporary publications luminal neovascularization, although reported in one study, was found to be negligible: vasculature originating from adventitial *vasa vasorum* exceeds luminal vessels 28 times [127]. This intimal neovasculature exclusively terminates in the outer *tunica intima* of the atherosclerotic human coronary artery, just above the internal elastic lamina, [113,116,123,127-131]. A comparable pattern of coronary outer *tunica intima* neovascularization has been demonstrated in a porcine model of coronary atherosclerosis [132].

Now, we shall enumerate the facts:

(1) Normal coronary DIT is an avascular compartment, receiving blood constituents through diffusion from the arterial lumen;

(2) Normal outer DIT is the most distant compartment from the arterial lumen and adventitial *vasa vasorum*. Therefore, the probability of diffusion to this depth of some blood constituents including LDL-C particles is very low;

(3) The outer avascular *tunica intima* of normal and atherosclerotic coronary is always reached by proteoglycan biglycan, which has a high capacity for selective binding of lipoproteins;

(4) In normal coronary artery, biglycan of the outer DIT does not have direct contact with blood, and interaction with LDL-C is prevented by diffusion distance and the properties of this molecule (20 nm);

(5) In coronary atherosclerosis, the outer layers of DIT become exclusively neovascularized, and biglycan comes into direct contact with blood lipoproteins.

If the above statements stand, a simple conclusion can be reached: in coronary atherosclerosis, biglycan of the outer DIT should extract and retain LDL-C particles from newly formed capillary beds, which are known to be very permeable [133,134]. This mechanism does not require any conditioning or complicated explanatory pathways. Furthermore, as we know from observations, lipid accumulation during early stages of coronary atherosclerosis always begins in the outer layers of the coronary DIT [61,62,64,67].

The assumption that neovascularization of the outer *tunica intima* is the first step in pathogenesis results in a hypothesis that produces the simplest explanations: (1) an initial deep localization of lipid deposition in the *tunica intima*, (2) a certain probability of coronary lipid deposition and atherosclerosis development when blood LDL levels are normal if pathological neovascularization has occurred, owing to LDL-C accessibility for contact with previously avascular structures (biglycan, which has affinity to LDL-C, and should extract it regardless of LDL-C levels); (3) more probable lipid deposition and disease contraction at high blood LDL levels; (4) probability of coronary atherosclerosis development after high LDL levels are lowered through the use of drugs, as neovascularization has already occurred and LDL-C particles appear in direct contact with previously avascular structures (biglycan, which has affinity to LDL-C and should extract it regardless LDL-C levels). At this point in the analysis, neovascularization of the coronary *tunica intima* appears as a cause of coronary atherosclerosis. Therefore, it logically follows that since the presence of LDL-C in plasma is a fundamental metabolic requirement for humans [135], theoretically there is no “safe LDL-C level” that would be 100% certain to prevent coronary atherosclerosis if intimal neovascularization has already occurred.

Therefore, the model predicts that if the coronary intima became vascularized, lipoproteins would be extracted and retained by intimal proteoglycan biglycan even if blood LDL levels were normal. However, lipoprotein extraction and deposition will be faster if LDL levels are high. These model predictions have been confirmed by clinical observations. Therefore, contrary to the accepted model, the author’s hypothesis suggests a different cause of the disease, and the opposite route for invasion of atherogeneic lipoproteins into the coronary *tunica intima*.

It is plausible that other intimal components, which were expressed and stored in the avascular environment, would interact with blood lipoproteins in the neovascularized environment. Hypothetical affinity to and binding of lipoproteins could be the result of LDL-C availability and matrix modifications under oxygenized conditions [136].

The author’s hypothesis does not refute the contribution of lipoprotein deposition from the arterial lumen. It is known that such deposition occurs in normal aorta, although resulting in a different pattern [107]. However, in the author’s model, lipoprotein deposition from the arterial lumen becomes irrelevant. Let us just compare the probability of two events occurring (i.e. lipid deposition via two pathways): (1) lipoproteins travel from the arterial lumen through the endothelium and multiple cell/matrix layers to be deposited in the outer DIT; (2) lipoproteins exude into the outer DIT from newly formed capillary beds, which terminate directly into the outer DIT and are very permeable [133,134]. The greater likelihood of the second model is obvious. The same logic could be applied to infer a route of monocyte infiltration into the coronary intima.

In previous publications, a similar mechanism was suggested to contribute to progression of already formed coronary plaques and inflammation in advanced human coronary atherosclerosis [137-140]. However, all prior analyses stop short of suggesting that neovascularization of the outer *tunica intima* is the cause of the disease.

This suggested mechanism of pathology is not unique. The identical mechanism, involving neovascularization of a normally avascular tissue compartment, followed by lipoprotein deposition, is well known. Consider corneal lipid keratopathy. The cornea is normally an avascular compartment [141,142]. More than 50 years ago, Cogan and Kuwabara described cornea lipid keratopathy, consisting of lipid deposition followed by fatty plaque formation, as occurring only in corneal areas that have been previously neovascularized [143]. Furthermore, the authors pointed to morphological similarities between cornea lipid plaques and those in atherosclerosis, and suggested common pathogenesis [143]. In succeeding years, numerous reports reaffirmed a causal role of neovascularization in corneal lipid deposition and hence the main treatment modality has become the inhibition of neovascularization [141,142,144-153]. In addition, there is only a single clinical observation of lipid keratopathy without prior neovascularization [154], and a single experimental study that disputes the causal role of neovascularization in corneal lipid deposition [155]. Furthermore, it has been noted that a role of inflammation during this pathogenesis is limited to the induction of angiogenesis [152]. Lipoprotein levels in the aqueous humor are thought to be close to those in blood [156-161]. It is important to note that although the corneal *substantia propria* is separated from aqueous humor by only one cell layer of descemet epithelium, lipid depositions have never been observed prior to corneal neovascularization (except the one report mentioned above [154]). This strongly favors the model of lipids exuding from permeable neovasculature into the cornea proper, rather than a diffusion model.

The fact that a similar sequence of events that includes lipid deposition underlines pathogenesis of the unrelated corneal disease reinforces the suggested new hypothesis concerning mechanisms of coronary atherosclerosis.

Why does arterial *tunica intima* become neovascularized in the first place?

Early during life the *tunica intima* of human coronary arteries differentiates from a single-layer cell compartment into a multi-layer cellular structure (i.e. DIT) through proliferation of residual and medial cells, and probably through participation of blood-born cells. Intimal proliferation with increasing numbers of cells continues until approximately 30 years of age [68,72] and then maintains self-renewal in a controlled manner throughout life. The mechanisms that initiate this morphogenesis and control it later during life are unknown, but it can be concluded that cells in the coronary *tunica intima* possess inherently high proliferative capacity. During normal growth transformations the coronary DIT remains avascular, so its dimension (thickness) allows all intimal cells to receive sufficient oxygen and nutrients through diffusion from the arterial lumen.

If we were to choose one feature that would universally reflect the reaction of the arterial *tunica intima*, and particularly the coronary intima, to a variety of stimuli, injuring factors, and interventions in clinics and experiments, the answer is undoubted - it is intimal cell proliferation. Regardless of the nature and magnitude of stimuli/insults, cells that appear in the arterial intimal compartment (normal or artificial, e.g. [162-167]), always proliferate in response. Furthermore, it is known that the arterial *tunica intima* can develop two normal variant phenotypes: a one-cell lining and a multi-layered cellular compartment, i.e. DIT. The first phenotype is maintained in all small and most medium caliber arteries, but certain arterial segments (e.g. coronary) normally evolve into the second phenotype. Each intimal type can be maintained as stable phenotypes or produce excessive intimal cell proliferation. Multiple observations have demonstrated that cells participating in this morphogenesis can be of different origins. As to regulations directing normal and pathological morphogenesis, a shear stress was suggested as the major factor [168-178]. In addition, I hypothesized that the arterial blood-tissue interface itself (as a topological entity) contributes to this morphogenesis, and the enhanced proliferative capacity of the arterial intima is a reflection of phenotype selection [69,179] (though these statements do not suggest mechanisms of regulation). All observations demonstrate that intimal proliferation can be induced by a variety of stimuli and insults that are different in nature and magnitude, which suggests that these stimuli and insults act as non-specific factors triggering preexisting regulation for proliferative morphogenesis. The ability of the arterial intima, and particularly coronary intima, to slip into proliferative morphogenesis was described as a genetic predisposition, which could manifest in “a hyperplastic vasculomyopathy” [180].

Therefore, cells in the coronary *tunica intima* respond by proliferating to any stimuli, exogenous or endogenous. An increase in cell numbers inevitably expands intimal thickness, which occurs with aging [119,181]. Expanded intimal thickness impairs diffusion of oxygen, as diffusion is inversely proportional to the square of the distance. Insufficient oxygen diffusion would inevitably result in hypoxia, specifically of cells in the outer DIT, because this tissue compartment is the most distant from the lumen and adventitial *vasa vasorum*[182].

What would happen when the coronary DIT becomes larger owing to cell proliferation or excessive matrix deposition (I did not mention a possible participation of intimal matrix before because there are few facts describing this pathway)? A straightforward answer was given by Gladstone Osborn: “When the intima of the coronary artery exceeds a certain thickness parts must either die or develop secondary blood supply” [183]. Since tissue hypoxia is a known inducer of angiogenesis and pathological neovascularization [184,185], neovascularization of the outer compartment of disease coronary DIT from adventitial *vasa vasorum* must follow coronary DIT expansion. The author agrees with Geiringer’s assertion that “…intimal vascularization is a function of intimal thickness and not of atherosclerosis” [105]. Furthermore, the author’s deduction from the above is that intimal proliferation/thickening and neovascularization are the causes of coronary atherosclerosis.

Therefore, it is hypothesized herein that proliferation of intimal cells initiates atherosclerosis. This is not a new model. This mechanism was suggested some time ago, although omitting subsequent neovascularization of coronary DIT [186-192]. However, the viewpoint that intimal cell proliferation is the beginning of atherosclerosis [186-192] was superseded by the currently endorsed hypothesis, which asserts that arterial intimal proliferation is an event secondary to lipid/macrophage penetration and inflammation [2,3,5,6,193]. Reflecting on the convenient hypothesis, the current classification of atherosclerosis excludes a variety of arterial pathologies characterized by intimal cell proliferation [194]. However, the currently endorsed hypothesis is based on an incorrect perception of coronary artery morphology. DIT enlargement and subsequent neovascularization were not recognized as initiators of the disease, and this view does not acknowledge outer lipid deposition as paradoxical. The currently endorsed model, based on invasion of lipoproteins from the coronary lumen, is very unlikely in the light of preceding DIT neovascularization. In the model outlined herein, neovascularization of the deep layers of DIT from the *vasa vasorum* makes initial outer intimal lipid deposition logical not paradoxical. Neovascularization of the previously avascular deep layers of coronary DIT, resulting in availability of blood lipoproteins to be extracted and retained by the DIT matrix, explains controversies regarding normal LDL-C levels (spontaneous or drug-modulated) and risks for coronary atherosclerosis.

**Figure 6:**
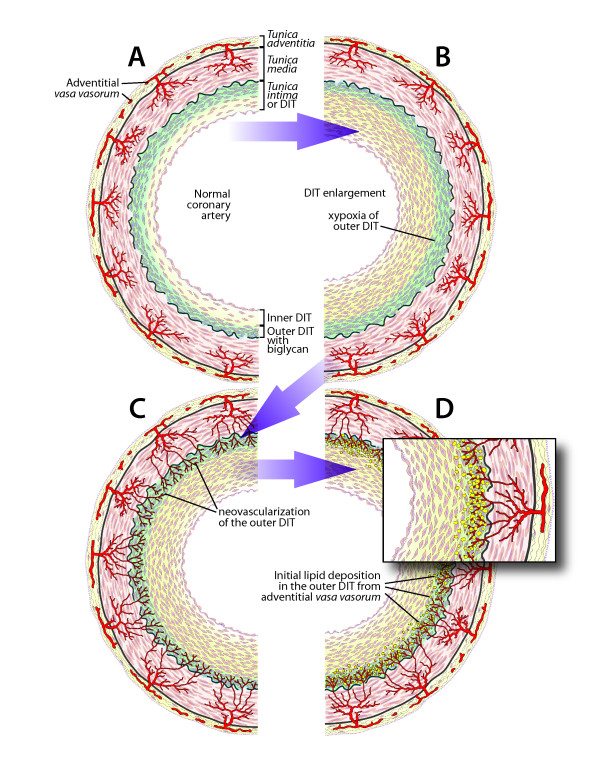
**Schematic representations of the mechanism of CA.a** – normal coronary artery. Coronary *tunica intima* forms DIT with biglycan accumulations in the outer DIT, which is most distant from the arterial lumen. **b** – DIT enlarged by cell proliferation and matrix production. Cells in the outer DIT underwent hypoxia due to increased diffusion distance. **c** – neovascularization of the outer DIT from adventitial *vasa vasorum*. Newly formed vessels are highly permeable. **d** – biglycan of the outer DIT comes in direct contact with blood LDL-C, which facilitates binding, retention and deposition of LDL-C in outer DIT, while inner DIT is free from lipoproteins. This schematic stage **d** corresponds to fatty streak Grade 1 and Grade 2 in the Nakashima *et al*. study [62]. Please note, in the schematic of a normal coronary artery (**a**), the number of DIT layers shown is less than my estimation in the text. This alteration was necessary to present half of the arterial circumference and emphasize DIT enlargement at the same time in the picture.

The suggested hypothesis can be presented in the following schematics (Figure 6):

##  Summary

(1) A hypotheses underlining our efforts to approach coronary atherosclerosis must be consistent with undisputed facts concerning the subject. Furthermore, a hypothesis should incorporate logical evaluation, and not contradict established and proven concepts in biology and medicine without well-grounded reasons.

(2) Atherosclerosis occurs in arteries with normal DIT, while sparing the rest of arterial bed. However, while normal DIT exists in numerous arteries [120,194], some of these are never affected by atherosclerosis; coronary arteries are almost always the target. On logical grounds, an arterial disease that never affects some arteries but usually affects certain others is not systemic.

(3) Coronary atherosclerosis is not an inflammatory disease, as multiple clinical trials demonstrate no correlation between anti-inflammatory therapies and risk of disease.

(4) High LDL levels are not a fundamental cause of coronary atherosclerosis, as lowering such levels protects only 30-40% of those at risk. Furthermore, humans and animals with normal LDL levels can suffer from coronary atherosclerosis.

(5) Neovascularization of the normally avascular DIT is the obligatory condition for coronary atherosclerosis development. This neovascularization originates from adventitial *vasa vasorum* and vascularizes the outer part of the coronary DIT, where LDL deposition initially occurs.

(6) It is suggested that excessive cell replication in DIT is a cause of DIT enlargement. Participation of enhanced matrix deposition is also plausible. An increase in DIT dimension impairs nutrient diffusion from the coronary lumen, causing ischemia of cells in the outer part of coronary DIT.

(7) Ischemia of the outer DIT induces angiogenesis and neovascularization from adventitial *vasa vasorum*. The newly formed vascular bed terminates in the outer part of the coronary DIT, above the internal elastic membrane, and consists of permeable vasculature.

(8) The outer part of the coronary DIT is rich in proteoglycan biglycan, which has a high binding capacity for LDL-C. While in avascular DIT, biglycan has very limited access to LDL-C due to diffusion distance and LDL-C properties; after neovascularization of the outer DIT, proteoglycan biglycan acquires access to LDL-C particles, and extracts and retains them.

(9) Initial lipoprotein influx and deposition occurs from the neovasculature originating from adventitial *vasa vasorum* - and not from the arterial lumen.

(10) Although lipoprotein deposition in the outer part of the coronary DIT is the earliest pathological manifestation of coronary atherosclerosis, intimal neovascularization from adventitial *vasa vasorum* must precede it.

Therefore, in the coronary artery *tunica intima*, a previously avascular tissue compartment becomes vascularized. All other tissue compartments are developed (both phylogenetically and ontogenetically) with constant exposure to capillary bed and blood, therefore their tissue components were selected not to bind LDL. This is why atherosclerosis is mostly limited to the coronary arteries. To my knowledge the only other example – the avascular cornea – shows the same lipid deposition after neovascularization.

The author does not claim that his hypothesis offers an immediate solution. Intimal cell proliferation, producing DIT and its later expansion, is cell hyperplasia, meaning that newly arrived cells are similar to normal residual cells, making systemic targeting very difficult. While the author strongly believes that intimal neovascularization is the crucial step in the pathogenesis of coronary atherosclerosis, there are obvious concerns about angiogenesis inhibition in a heart with an already jeopardized myocardial blood supply. The author does not intend to suggest an immediate solution. The goal was to evaluate the hypothesis and the perceptions that we exercise in approaching coronary atherosclerosis logically and factually, and to offer a more coherent model. Furthermore, the intent was to underline paradoxical observations that could provide new insights into mechanisms of the disease. Atherosclerotic plaque growth and rupture are not paradoxical but anticipated events. In contrast, initial lipid deposition in outer layers of DIT with no deposition in inner layers is a paradoxical observation, and requires an explanatory model that differs from the accepted one. However, to recognize the paradox, correct perception of the coronary artery structure, where pathology occurs, must not be distorted by incorrect illustrations and verbal descriptions. When we name or depict things incorrectly, often just for nosological reasons, the incorrect perception of events may persist in spite of growing knowledge, impeding our attempts to discover the truth.

## Conflict of interest

The author declares that he has no competing interests.

## Author’s contribution

VMS conducted all the work involved in preparing and writing this paper.
